# Prediction of Ovarian Hyperstimulation Syndrome in Patients Treated with Corifollitropin alfa or rFSH in a GnRH Antagonist Protocol

**DOI:** 10.1371/journal.pone.0149615

**Published:** 2016-03-07

**Authors:** Georg Griesinger, Pierre J. M. Verweij, Davis Gates, Paul Devroey, Keith Gordon, Barbara J. Stegmann, Basil C. Tarlatzis

**Affiliations:** 1 Department of Reproductive Medicine and Gynecological Endocrinology, University Hospital of Schleswig-Holstein, Campus Luebeck, Luebeck, Germany; 2 MSD BV, Oss, The Netherlands; 3 Merck & Co., Inc., Kenilworth, NJ, United States of America; 4 Center for Reproductive Medicine, Universitair Ziekenhuis Brussel, Brussels, Belgium; 5 1st Department of Obstetrics and Gynecology, Papageorgiou Hospital, School of Medicine, Aristotle University of Thessaloniki, Thessaloniki, Greece; Inner Mongolia University, CHINA

## Abstract

**Study Question:**

What is the threshold for the prediction of moderate to severe or severe ovarian hyperstimulation syndrome (OHSS) based on the number of growing follicles ≥ 11 mm and/or estradiol (E_2_) levels?

**Summary Answer:**

The optimal threshold of follicles ≥11 mm on the day of hCG to identify those at risk was 19 for both moderate to severe OHSS and for severe OHSS. Estradiol (E_2_) levels were less prognostic of OHSS than the number of follicles ≥ 11 mm.

**What Is Known Already:**

In comparison to long gonadotropin-releasing hormone (GnRH) agonist protocols, the risk of severe OHSS is reduced by approximately 50% in a GnRH antagonist protocol for ovarian stimulation prior to in vitro fertilisation (IVF), while the two protocols provide equal chances of pregnancy per initiated cycle. Nevertheless, moderate to severe OHSS may still occur in GnRH antagonist protocols if human chorionic gonadotropin (hCG) is administered to trigger final oocyte maturation, especially in high responder patients. Severe OHSS following hCG trigger may occur with an incidence of 1–2% in a relatively young (aged 18 to 36 years) IVF population treated in a GnRH-antagonist protocol.

**Study Design, Size, Duration:**

From the Engage, Ensure and Trust trials, in total, 2,433 women who received hCG for oocyte maturation and for whom the number of follicles ≥ 11 mm and the level of E_2_ on the day of hCG administration were known were included in the analyses.

**Participants/Materials, Setting, Methods:**

The threshold for OHSS prediction of moderate and severe OHSS was assessed in women treated with corifollitropin alfa or daily recombinant follicle stimulation hormone (rFSH) in a gonadotropin-releasing hormone (GnRH)-antagonist protocol. Receiver operating characteristics curve analyses for moderate to severe OHSS and severe OHSS were performed on the combined dataset and the sensitivity and specificity for the optimal threshold of number of follicles ≥ 11 mm, E_2_ levels on the day of (hCG), and a combination of both, were determined.

**Main Results and the Role of Chance:**

The optimal threshold of follicles ≥ 11 mm on the day of hCG to identify those at risk of moderate to severe OHSS was 19 (sensitivity and specificity 62.3% and 75.6%, respectively) and for severe OHSS was also 19 (sensitivity and specificity 74.3% and 75.3%, respectively). The positive and negative predictive values were 6.9% and 98.6%, respectively, for moderate to severe OHSS, and 4.2% and 99.5% for severe OHSS.

**Limitations, Reasons for Caution:**

This was a retrospective analysis of combined data from three trials following ovarian stimulation with two different gonadotropins.

**Wider Implications of the Findings:**

For patients with 19 follicles or more ≥11 mm on the day of hCG, measures to prevent the development of OHSS should be considered. Secondary preventive measures include cycle cancellation or coasting, use of a GnRH agonist to trigger final oocyte maturation in place of hCG and a freeze all strategy.

**Trial Registration:**

ClinicalTrials.gov NCT00702845

NCT00696800

NCT00696878

## Introduction

Ovarian hyperstimulation syndrome (OHSS) is a potentially fatal complication of ovarian stimulation as part of assisted reproduction. The incidence of severe OHSS has been reported to range from 0.7 to 1.7% per initiated cycle with hospitalization for OHSS occurring in 0.9 to 1.4% of IVF cycles [1]. Severe OHSS is predicted to affect over 6000 patients per year in the United States and Europe [2].

The primary, independent risk factors associated with the development of OHSS in a gonadotropin-releasing hormone (GnRH)-antagonist protocol have recently been identified as low basal follicle-stimulating hormone (FSH), high peak estradiol (E_2_) after ovarian stimulation and a high number of growing follicles [3].

Except for rare cases with an abnormal genetic background, OHSS only develops after ovarian stimulation and multifollicular recruitment [4]. The triggering agent of OHSS is human chorionic gonadotropin (hCG), either administered exogenously for induction of final oocyte maturation and/or derived endogenously in the case of pregnancy. Accordingly, two types of OHSS are distinguished: the early-onset, which is limited by the duration of exogenous hCG activity in the circulation after administration (e.g., onset and full manifestation of OHSS within 9 days after hCG administration); and the late-onset, which develops ten days or later after hCG administration in the case of pregnancy [5].

In comparison to long GnRH agonist protocols, the risk of severe OHSS is reduced by approximately 50% in a GnRH antagonist protocol for ovarian stimulation prior to in vitro fertilisation (IVF) or intra-cytoplasmic sperm injection, while the two protocols provide equal chances of pregnancy per initiated cycle [6]. Nevertheless, moderate to severe OHSS may still occur in GnRH antagonist protocols if hCG is administered to trigger final oocyte maturation, especially in high responder patients. Severe OHSS following hCG trigger may occur with an incidence of 1–2% in a relatively young (aged 18 to 36 years) IVF population treated in a GnRH-antagonist protocol [3].

Identification of women at risk of OHSS is important so that they will primarily not be treated with long GnRH-agonist protocols and high FSH doses, while in those subjects still presenting with a high ovarian response, individualised secondary preventive measures can be applied.

The objective of the current study was to identify a threshold for the prediction of OHSS based on the number of growing follicles ≥11 mm and/or E_2_ levels induced by treatment with either corifollitropin alfa or daily rFSH in a GnRH antagonist protocol. For this purpose, prospectively collected data from three phase III trials performed according to Good Clinical Practice standards were used [7–9]. This large database of phase III trial data was also used to validate the previously reported threshold for the number of follicles ≥11 mm to predict OHSS [10].

## Material and Methods

### Trials included

The threshold for OHSS prediction based on the number of growing follicles and serum E_2_ concentrations was assessed in women with an indication for ovarian stimulation, treated with corifollitropin alfa and rFSH in a GnRH antagonist protocol in three phase III trials (Engage, Ensure and Trust). Details of these trials, Engage [8], Ensure [7], and Trust [9], have been previously reported.

Briefly, Engage and Ensure were double-blind, double-dummy randomized controlled trials that compared the efficacy of a single injection of corifollitropin alfa with daily injections of rFSH in a GnRH antagonist protocol. Trust was an open, uncontrolled trial that evaluated the safety and tolerability of repeated cycles (up to three per patient) with a single injection of corifollitropin alfa in a GnRH antagonist protocol. All three trials included in this analysis were conducted in accordance with principles of Good Clinical Practice and were approved by the appropriate institutional review boards and regulatory agencies, and written informed consent was provided by all subjects.

### Study population

In the Engage trial, women aged 18–36 years with a body weight of >60 to ≤90 kg and body mass index (BMI) 18–32 kg/m^2^ received either a single dose of corifollitropin alfa 150 μg (n = 755) or 7 daily injections of rFSH 200 IU (n = 751) for the first 7 days of COS followed by daily rFSH [8]. Women aged 18–36 years with a body weight ≤60 kg and a BMI 18–32 kg/m^2^ received either a single dose of corifollitropin alfa 100 μg (n = 268) or 7 daily injection of rFSH 150 IU (n = 129) for the first 7 days of COS followed by daily rFSH in the Ensure trial [7]. In the Trust trial, women aged 18–39 years with a body weight of >60 kg and a BMI 18–29 kg/m^2^ received a single dose of corifollitropin alfa 150 μg (n = 682) for the first 7 days of COS followed by daily rFSH. For the current analysis, only the first cycle of every patient was considered.

In all three trials, women who had a history of ovarian hyper-response to ovarian stimulation (more than 30 follicles ≥11 mm) or OHSS, polycystic ovary syndrome or a basal antral follicle count of more than 20 on ultrasound (<11 mm, both ovaries combined) were excluded from participation.

### Study design

Women started ovarian stimulation on day 2 or 3 of their menstrual cycle with corifollitropin alfa treatment (or daily rFSH for Engage and Ensure) in the Engage, Ensure and Trust trials. From stimulation day 8 onwards, treatment was continued with a daily subcutaneous dose of ≤ 200 IU rFSH (Engage and Ensure) or ≤225 IU rFSH/human menopausal gonadotrophin (Trust) up to and including (optional in Trust) the day of hCG administration. Starting on stimulation day 5 and up to and including the day of hCG administration, GnRH antagonist ganirelix was administered to prevent premature LH surges. Final oocyte maturation was induced with urinary hCG (10,000 IU or 5000 IU, at the discretion of the physician, in the case of a high ovarian response; Engage, Ensure and Trust) or rhCG 250 μg (Trust) as soon as three follicles ≥17 mm were observed by ultrasound scan or the next day.

The dose of rFSH, in the daily rFSH treatment arms, could be reduced from day 6 (Engage and Ensure) or day 8 (Trust) onwards in the case of a too-high ovarian response. The investigator could reduce the dose of rFSH (dose tapering) or withhold rFSH administration for a maximum of 3 days (coasting) up to and including the day of hCG administration.

If the ovarian response was too high, in the opinion of the investigator, the cycle could be cancelled at any time. If there was a risk of OHSS (>30 follicles ≥11 mm), hCG administration was withheld and the treatment cycle was cancelled. The maximum total duration of stimulation allowed within the studies was 19 days.

Serum E_2_ concentrations were measured at a central laboratory (Waltrop Organon Development GmbH, Waltrop, Germany).

### Statistical analysis

OHSS was graded as mild, moderate or severe, by World Health Organization criteria[11] based on the investigator’s description on the adverse events case report form. Three endpoints were defined: OHSS of any grade, moderate to severe OHSS and severe OHSS (severe OHSS was included in all three endpoints).

Only women who received hCG to trigger oocyte maturation and for whom the number of follicles ≥11 mm and the E_2_-level on the day of hCG were known were included in the analyses. Ninety-three subjects (3.7%) who received hCG were excluded because of missing data for follicles and/or E_2_ levels on the day of hCG.

Initially, logistic regression was performed for dependent variables moderate and severe OHSS and severe OHSS and with these covariates: number of follicles ≥11 mm on the day of hCG or E_2_ levels on the day of hCG, treatment group, and trial. As regression analysis indicated no effects of treatment group and trial on the incidence of OHSS, data were pooled for treatment groups and the three trials.

Each OHSS endpoint was analyzed using a logistic regression model with a covariate for the number of follicles ≥11 mm on the day of hCG (or E_2_ level on the day of hCG, respectively). The associated receiver operating characteristics (ROC) curve was plotted and the area under the curve (AUC, or c-statistic) was calculated.

A threshold value for the number of follicles ≥11 mm on the day of hCG (or E_2_ level on the day of hCG, respectively) was based on the 'optimal' point on the ROC curve. This is the point that is closest in distance to the upper left hand corner where sensitivity and specificity are equal to one and, hence, provides the best trade-off between sensitivity and specificity.

Sensitivity, specificity and positive and negative predictive values at the optimal threshold were determined. Additionally, these test characteristics were calculated for previously published thresholds for moderate to severe OHSS and severe OHSS in patients undergoing GnRH-antagonist stimulation for IVF [10].

## Results

Overall, 2585 women in the Engage, Ensure and Trust trials were treated with either corifollitropin alfa or rFSH. In total, 2526 women received hCG for oocyte maturation of whom 2433 were assessed for the number of follicles ≥11 mm on day of hCG administration and E_2_ evaluation. In these women, a total of 136 cases of OHSS were observed: 67 were graded as mild, 34 as moderate and 35 as severe.

In the Engage, Ensure and Trust trials, the dose of rFSH was reduced in 24%, 22%, and 7% of women, respectively, and the cycle was cancelled in 0.3%, 0% and 0%, respectively, due to risk of OHSS.

### Characteristics of women included in the analysis from the three trials

Characteristics of women included in the current analysis of prediction of OHSS in the Engage, Ensure and Trust trials are described in Table 1. Their mean age was similar across the three trials: 31.5 years in Engage, 31.0 years in Ensure, and 32.9 years in Trust. The mean number of follicles ≥11 mm on the day of hCG was also similar: 14.9, 14.2 and 14.5 in the in Engage, Ensure and Trust trials, respectively, and mean E_2_ levels on the day of hCG were 5323, 5223 and 5595 pmol/L, respectively. The serum E_2_ levels on the day of hCG varied considerably and did not correlate highly with the number of follicles (correlation coefficient 0.49). In total, 83%, 84% and 66% of women in the Engage, Ensure and Trust trials, respectively, received 10,000 IU hCG, and 17%, 16% and 9%, respectively, received 5,000 IU hCG. The mean number of oocytes retrieved was 13.5 in Engage, 12.4 in Ensure and 12.3 in Trust. In the Engage, Ensure, and Trust studies, respectively, the numbers of good quality (Grades 1 and 2) embryos obtained were 4.5, 3.2 and 3.0, of which 1.3, 1.2, and 1.3 were transferred. The ongoing pregnancy rates were 39.0% in Engage, 28.8% in Ensure and 23.2% in Trust.

**Table 1:** Patient characteristics.

	Engage N = 1405	Ensure N = 389	Trust N = 639	Total N = 2433
Age (years), mean (SD)	31.5 (3.3)	31.0 (3.1)	32.9 (3.6)	31.8 (3.4)
BMI (kg/m2), mean (SD)^a^	24.8 (2.7)	20.6 (1.5)	24.2 (2.4)	24 (2.9)
AFC, mean (SD)^b^	12.4 (4.5)	11.1 (4.4)	10.8 (4.7)	11.8 (4.6)
Dose reductions of rFSH from day 8 onwards, %^c^	46.1	37.1	90.3	56.7
Oocytes retrieved, mean (SD)	13.5 (7.4)	12.4 (6.9)	12.3 (7.0)	13.0 (7.2)
Fertility characteristics				
Primary infertility, %	53.0	61.4	57.0	55.4
Duration of infertility (years), mean (SD)^d^	3.3 (2.4)	3.2 (2.2)	3.7 (2.9)	3.4 (2.5)
Dose of hCG administered^e^				
10,000 IU, %	83.2	83.8	66.4	78.8
5000 IU, %	16.8	16.2	8.8	14.6
rhCG 250 μg, %	0.0	0.0	24.9	6.5
Day of hCG				
Follicles ≥11 mm, mean (SD)	14.9 (6.6)	14.2 (6.4)	14.5 (6.5)	14.7 (6.5)
Serum E_2,_ pmol/L, mean (SD)	5323.1 (3241.4)	5222.7 (3272.9)	5595.0 (3393.0)	5378.5 (3288.3)
Serum FSH, IU/L, mean (SD)	12.1 (3.1)	11.6 (3.0)	13.6 (3.7)	12.4 (3.3)
Serum LH, IU/L, mean (SD)	1.6 (1.7)	1.5 (1.7)	1.5 (1.9)	1.6 (1.8)
Serum P, nmol/L, mean (SD)	3.1 (1.8)	2.5 (1.5)	4.1 (23.4)	3.3 (12.1)

^a^6 missing body mass index (BMI) values;

^b^64 missing antral follicle count (AFC);

^c^based on 2129 patients receiving rFSH from day 8 onwards;

^d^2 missing durations;

^e^3 other.

### Prediction of moderate to severe OHSS

**Fig 1:**
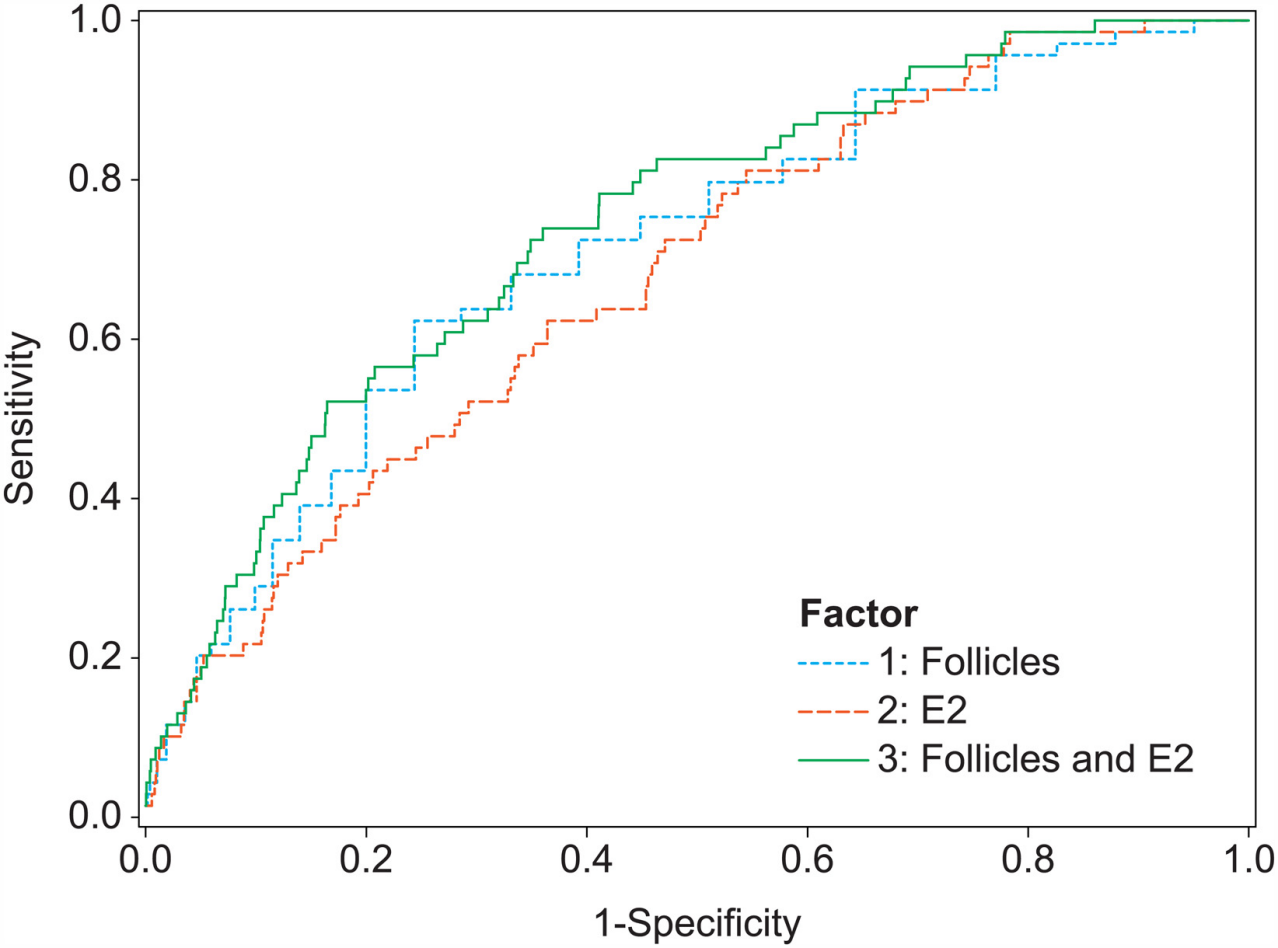
ROC curves for the prediction of moderate to severe OHSS (69 cases) based on the number of follicles ≥11 mm on the day of hCG, the E_2_ level on the day of hCG or both. AUCs: 0.728 (follicles); 0.678 (E_2_); 0.739 (follicles and E_2_).

In total, 69 women (2.8%) had moderate to severe OHSS. The ROC curves for two predictors are shown in Fig 1.

The AUC for the number of follicles ≥11 mm on the day of hCG was 0.728. The association with moderate to severe OHSS was statistically significant (P<0.0001, Table 2). For the prediction of moderate to severe OHSS, the optimal threshold was 19 follicles. The sensitivity and specificity were 62.3% and 75.6%, respectively. The percentage of subjects at ‘higher risk’ that actually experienced moderate to severe OHSS was 6.9% (positive predictive value, Table 3).

**Table 2:** Odds ratios for moderate to severe OHSS and severe OHSS for the number of follicles ≥11 mm and the E_2_ level on the day of hCG.

OHSS	Predictor(s)	Odds ratio^a^	95% CI	P-value	AUC^b^
Moderate to severe	Follicles ≥11 mm	1.113	1.081, 1.147	<0.0001	0.728
Moderate to severe	E_2_ level	1.165	1.106, 1.227	<0.0001	0.678
Moderate to severe	Follicles ≥11 mmand E_2_ level	1.090	1.026, 1.157	0.0049	0.739
Severe	Follicles ≥11 mm	1.105	1.064, 1.148	<0.0001	0.769
Severe	E_2_ level	1.190	1.115, 1.270	<0.0001	0.731
Severe	Follicles ≥11 mmand E_2_ level	1.129	1.049,1.216	0.0013	0.782

^a^Per additional follicle ≥ 11 mm or E_2_ increase of 1000 pmol/L; based on logistic regression model with the number of follicles ≥ 11 mm on the day of hCG, the E_2_ level on the day of hCG or both.

^b^Area under the Receiver Operating Characteristic (ROC) curve.

**Table 3:** Diagnostic test characteristics for various rules to predict OHSS.

OHSS	Predictor(s)	Sensitivity	Specificity	Predictive Value		Threshold
				Positive	Negative	
Moderate to severe	Follicles ≥11 mm	62.3%	75.6%	6.9%	98.6%	≥19
Moderate to severe	E_2_ level	62.3%	63.6%	4.8%	98.3%	≥5900
Moderate to severe	Follicles ≥11 mm and E_2_ level	72.5%	65.1%	5.7%	98.8%	≥ -3.66^a^
Severe	Follicles ≥11 mm	74.3%	75.3%	4.2%	99.5%	≥19
Severe	E_2_ level	71.4%	64.7%	2.9%	99.4%	≥6100
Severe	Follicles ≥11 mm and E_2_ level	74.3%	74.1%	4.0%	99.5%	≥ -4.18^b^
Previously reported threshold in current data						
Moderate to severe	Follicles ≥11 mm	82.6%	42.3%	4.0%	98.8%	≥13^c^
Severe	Follicles ≥11 mm	77.1%	71.1%	3.8%	99.5%	≥18^c^

^a^For linear predictor = -5.627 + 0.090 • follicles [≥ 11 mm] + 0.086 • E_2_ [1000 pmol/L];

^b^For linear predictor = -6.362 + 0.077 • follicles [≥ 11 mm] + 0.122 • E_2_ [1000 pmol/L];

^c^Papanikolaou [2006].

The AUC for the E_2_ level on the day of hCG was 0.678. The association with moderate to severe OHSS was statistically significant (P<0.0001, Table 2). For the prediction of moderate to severe OHSS, the optimal threshold was approximately 5900 pmol/L. In practice, a threshold of 6000 pmol/L (1634 pg/mL) may be used. The sensitivity and specificity were 62.3% and 63.6%, respectively. The percentage of subjects at ‘higher risk’ that actually experienced moderate to severe OHSS was 4.8% (positive predictive value, Table 3). Compared with the number of follicles, E_2_ was less prognostic for moderate to severe OHSS.

The sensitivity of the prediction of moderate to severe OHSS could be increased to 72.5% by including both factors simultaneously in a model. The AUC of that model was 0.739 (Fig 1, Table 2).

### Prediction of severe OHSS

**Fig 2:**
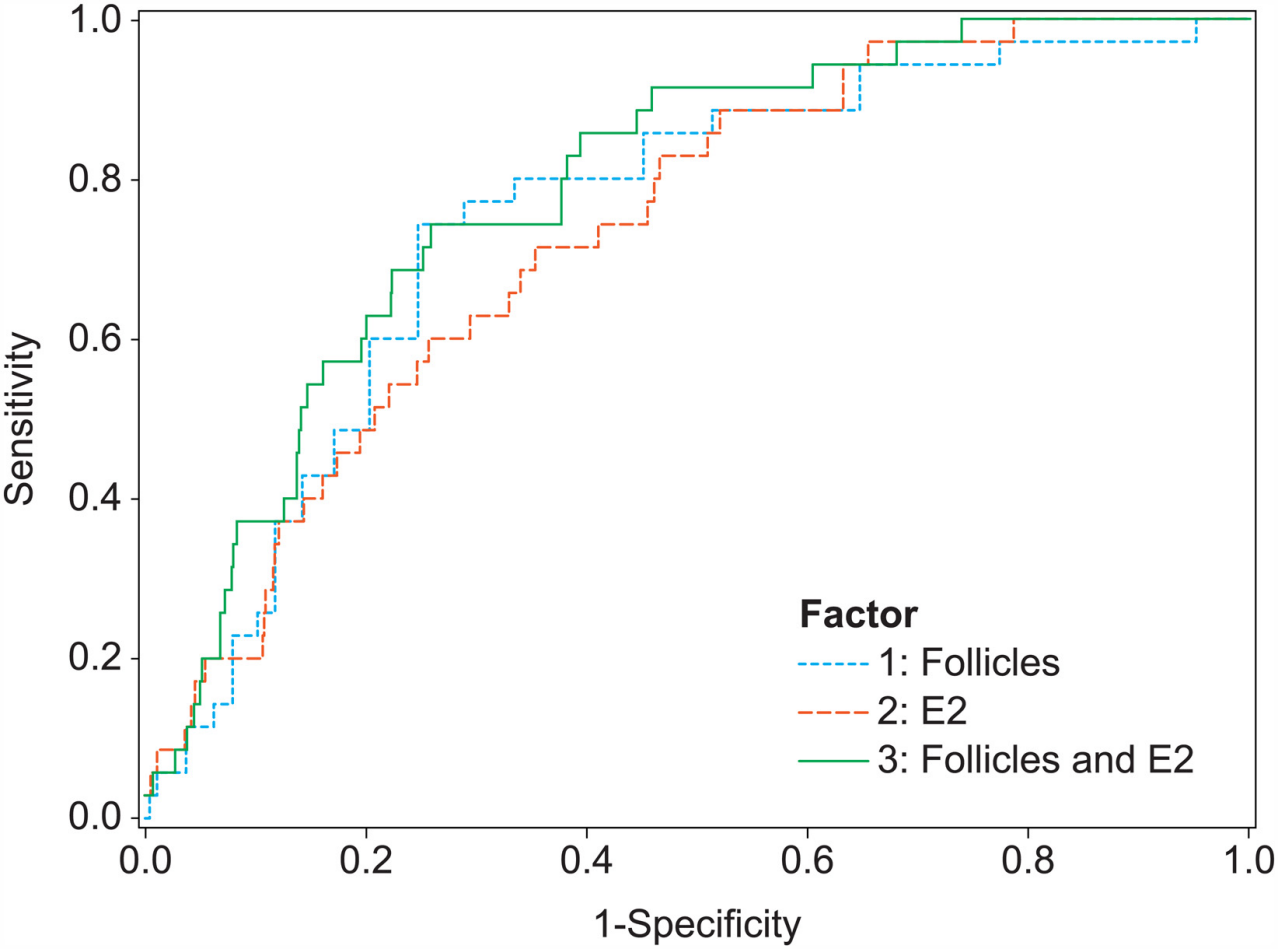
ROC curves for the prediction of severe OHSS (35 cases) based on the number of follicles ≥ 11 mm on the day of hCG, the E_2_ level on the day of hCG or both. AUCs: 0.769 (follicles); 0.731 (E_2_); 0.782 (follicles and E_2_).

In total, 35 subjects had severe OHSS (1.4%). The ROC curves for two predictors are shown in Fig 2.

The AUC for the number of follicles ≥11 mm on the day of hCG was 0.769. The association with severe OHSS was statistically significant (P<0.0001, Table 2). For the prediction of severe OHSS, the optimal threshold was 19 follicles, the same as for the prediction of moderate to severe OHSS. The sensitivity and specificity were 74.3% and 75.3%, respectively. The percentage of women at ‘higher risk’ that actually experienced severe OHSS was 4.2% (positive predictive value, Table 3).

The AUC for the E_2_ level on the day of hCG was 0.731. The association with severe OHSS was statistically significant (P<0.0001, Table 2). For the prediction of severe OHSS the optimal threshold was approximately 6100 pmol/L. Again, a threshold of 6000 pmol/L (1634 pg/mL) may be used in practice. The sensitivity and specificity were 71.4% and 64.7%, respectively. The percentage of women at ‘higher risk’ that actually experienced severe OHSS was 2.9% (positive predictive value, Table 3).

E_2_ was less prognostic for severe OHSS than the number of follicles.

The prediction of severe OHSS by including both factors in a model resulted in an AUC of 0.782 (Fig 2, Table 2).

Data for the prediction of OHSS of any grade are given in the Supporting Information, S1 Text, S1 Fig, and S1 and S2 Tables.

### Validation of previously reported threshold levels of number of follicles to predict OHSS in current data

Applying a threshold of ≥18 follicles ≥11 mm on the day of hCG [10] in the combined Ensure, Engage and Trust trials database yielded a sensitivity and specificity of 77.1% and 71.1%, respectively, for the prediction of severe OHSS (Table 3). For the prediction of moderate to severe OHSS, applying a threshold of ≥13 follicles ≥ 11 mm on the day of hCG [10] yielded a sensitivity and specificity of 82.6% and 42.3%, respectively (Table 3).

### Combining predictions of moderate to severe OHSS and severe OHSS

Searching for threshold values of predictors in order to identify women at increased risk for OHSS is helpful, but oversimplifies the problem. In practice, the risk of OHSS increases gradually with the number of follicles ≥11 mm and the E_2_ level on the day of hCG administration.

**Fig 3:**
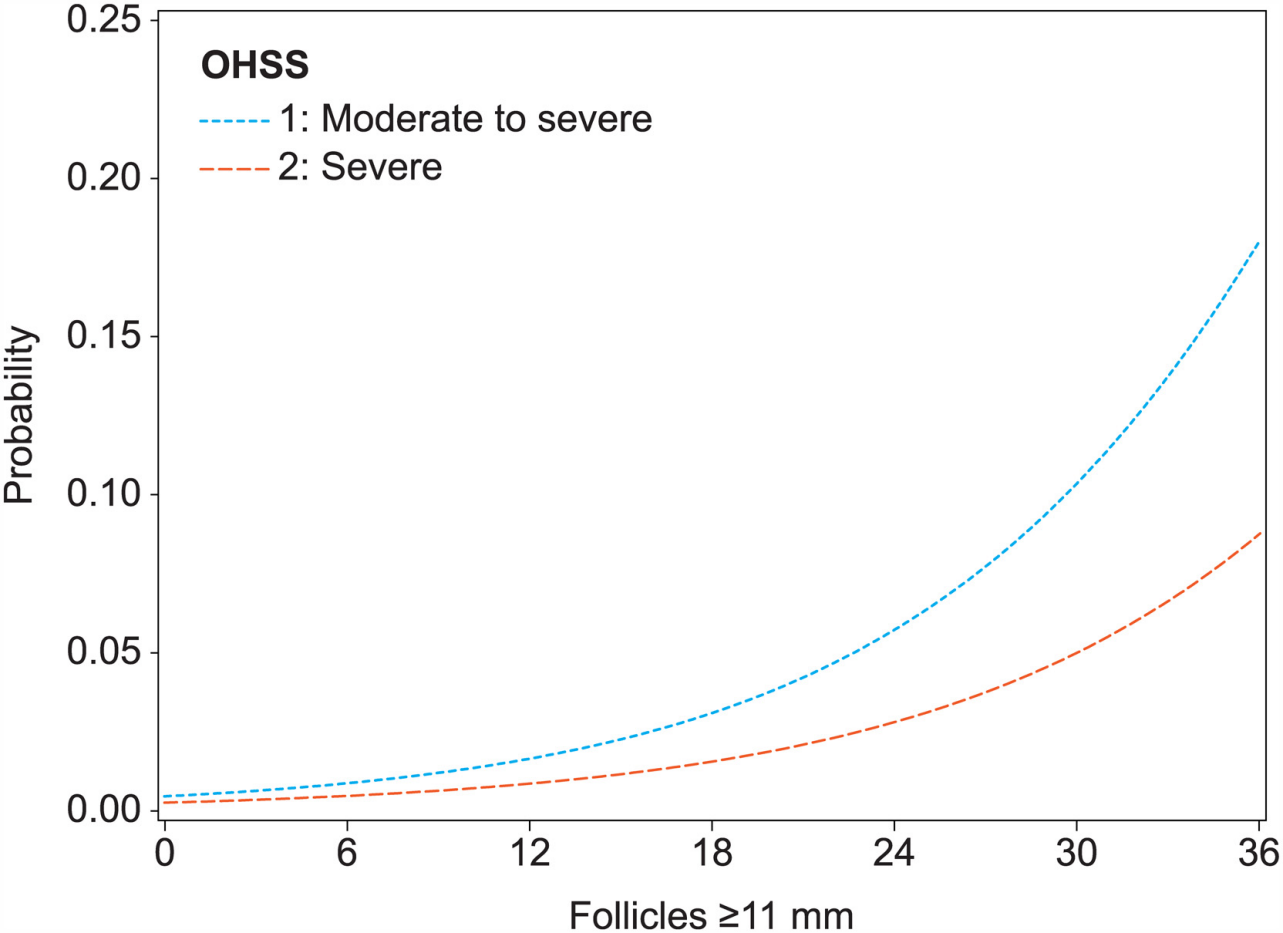
Probabilities of moderate to severe OHSS and severe OHSS associated with the number of follicles ≥11 mm on the day of hCG.

Fig 3 displays the predicted probabilities of the moderate to severe OHSS and severe OHSS endpoints depending on the number of follicles ≥11 mm. It can, for example, be seen that a woman with 16 follicles has a 2.3% probability for moderate to severe OHSS, and a 1.2% chance for severe OHSS. The probability for moderate OHSS, by subtraction, is 1.1%. Likewise, a patient with 19 follicles has a 1.6% chance for severe OHSS, and a 1.5% probability for moderate OHSS.

**Fig 4:**
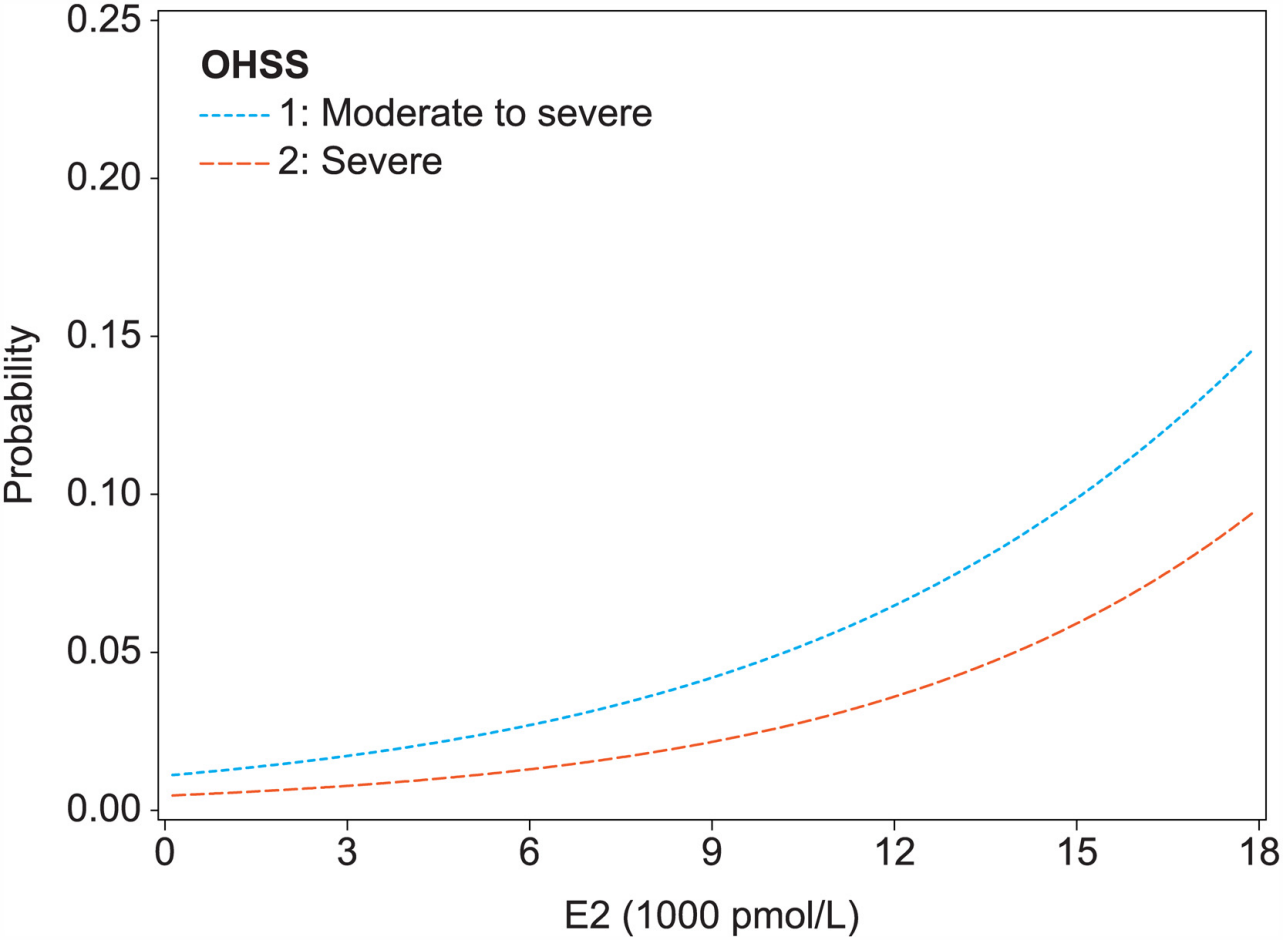
Probabilities of moderate to severe OHSS and severe OHSS associated with the E_2_ level on the day of hCG.

Fig 4 displays the predicted probabilities of the moderate to severe OHSS and severe OHSS endpoints depending on the E_2_ level on the day of hCG. The fit between predicted probabilities and actually observed proportions is addressed in Supporting Information figures (S2–S7 Figs).

## Discussion

This large, retrospective analysis of individual subject responses from 3 large phase III clinical trials suggests a total of 19 or more follicles ≥11 mm is associated with an increased risk of moderate to severe OHSS. Patients with less than 19 follicles were at low risk for severe OHSS (<1%); nevertheless, it is important to note that women with fewer than 19 follicles may also develop OHSS and therefore continued monitoring based on individual patient risk profile is required. In addition, despite that this analysis was the largest set of prospectively collected data on GnRH-antagonist stimulation available so far, the numbers were too small to separately predict early-onset and late-onset OHSS.

The numbers of follicles and serum E_2_ levels have been used previously to predict the risk of OHSS. In a rFSH GnRH antagonist protocol, Papanikolaou et al[10] showed that women with ≥13 follicles ≥11 mm on the day of hCG were at increased risk of developing moderate to severe OHSS, while women with ≥18 follicles ≥11 mm were at increased risk of developing severe OHSS. In a prospective randomized trial, Kwee et al reported that an antral follicle count of >14 had the highest sensitivity (82%) and specificity (89%) in predicting patients with a hyper response [12]. In a prospective observational study, the total number of medium/large-sized follicles before hCG administration was also shown to be an independent predictor of OHSS [13]. A high serum E_2_ concentration on the day of hCG trigger has also been suggested as a predictor of OHSS. However, high or rapidly rising E_2_ levels alone are unreliable and poor predictors of OHSS [2,5].

Predicting women who are at risk of developing OHSS was traditionally based on serum E_2_ levels. Most investigators consider an E_2_ level of 3000 pg/mL as the threshold to predict a risk of OHSS. However, applying an E_2_ threshold of 3000 pg/mL would have only predicted 1/3 of the total OHSS cases [2,10]. Even after applying the best threshold value of 2560 pg/mL, less than half of the severe cases were predicted (49% sensitivity, 77% specificity) [10].

In agreement with Papanikolaou et al [10], the number of follicles on the day of hCG was found to be superior to E_2_ levels in predicting severe OHSS. Predicting cases of OHSS using the number of follicles rather than E_2_ levels is likely to be superior because the release of vascular endothelial growth factor, which is implicated in the pathogenesis of OHSS, is related to the number of follicles rather than their E_2_ production [10].

It is also noteworthy that transvaginal sonography is considered standard care during ovarian stimulation and that it is a relatively easy-to-perform and therefore a robust methodology to monitor ovarian response. Accordingly, the information on the number of growing follicles is readily available for all clinicians practicing IVF, which is not necessarily the case for E_2_ measurements. Furthermore, variations in E_2_ assays between centers might impact on the performance of E_2_ as a predictor of OHSS.

Papanikolaou et al[10] achieved an 83% sensitivity rate with a specificity of 84% using the threshold of ≥18 follicles ≥11 mm on the day of hCG for the prediction of severe OHSS, and a 85.5% sensitivity rate with a specificity of 69% using the threshold of ≥13 follicles ≥11 mm on the day of hCG for the prediction of moderate to severe OHSS using their data. The lower sensitivity and specificity rates achieved following application of these criteria to the Engage, Ensure and Trust trials database (77.1% and 71.1%, respectively, for severe OHSS; 82.6% and 42.3%, respectively, for moderate to severe OHSS) is not unexpected, as a prediction rule usually performs worse in new data than in the data from which the rule was derived.

For patients with 19 follicles or more ≥11 mm on the day of hCG, measures to prevent the development of OHSS should be considered. Secondary preventive measures include: (1) cycle cancellation or coasting; (2) use of a GnRH agonist to trigger final oocyte maturation in place of hCG; and (3) a freeze-all strategy. Cycle cancellation is the most effective preventative measure, but it is also associated with emotional and financial stress [2,14]. The use of a GnRH agonist for triggering final oocyte maturation and ovulation in an antagonist cycle and subsequent fresh or frozen embryo transfer based on physician judgement is a default approach to reduce OHSS in at-risk patients[15,16]. The risk of late-onset OHSS can also be eliminated by freezing all embryos for subsequent embryo transfer in a natural cycle [17–19]. Furthermore, use of cabergoline [20,21] and adjuvant metformin [22] are efficacious in preventing the occurrence of moderate and severe OHSS, without a negative impact on pregnancy rates. *In vitro* maturation (IVM) is an emerging treatment option that eliminates OHSS [23–25]; however, IVM has been less successful than standard IVF with respect to outcomes [24,26,27]. Further study and use of these preventive measures may allow for the introduction of an OHSS-Free Clinic, as proposed by Devroey et al [28].

## Conclusions

This large retrospective analysis of phase III trial data suggests that 19 or more follicles ≥11 mm on the day of hCG leads to an increased risk for moderate to severe and severe OHSS. Serum E_2_ levels on the day of hCG administration performed less well than the number of follicles in predicting OHSS. Preventive measures should be considered for women with 19 or more follicles ≥11 mm on the day of oocyte maturation following ovarian stimulation with gonadotropins.

## Supporting Information

S1 Fig ROC curves for the prediction of OHSS of any grade (136 cases) based on the number of follicles ≥ 11 mm on the day of hCG, the E2 level on the day of hCG or both. AUCs: 0.720 (follicles); 0.696 (E2); 0.744 (follicles and E2).
(DOCX)

S2 Fig Observed proportions and expected probabilities for OHSS of any grade associated with the number of follicles ≥ 11 mm on the day of hCG. Dots represent subgroups with n≥50; circles represent smaller subgroups.
(DOCX)

S3 Fig Observed proportions and expected probabilities for OHSS of any grade associated with the E2 level on the day of hCG. Dots represent subgroups with at least 50 subjects; circles represent smaller subgroups.
(DOCX)

S4 Fig Observed proportions and expected probabilities for moderate to severe OHSS associated with the number of follicles ≥ 11 mm on the day of hCG. Dots represent subgroups with n≥50; circles represent smaller subgroups.
(DOCX)

S5 Fig Observed proportions and expected probabilities for moderate to severe OHSS associated with the E2 level on the day of hCG. Dots represent subgroups with n≥50; circles represent smaller subgroups.
(DOCX)

S6 Fig Observed proportions and expected probabilities for severe OHSS associated with the number of follicles ≥ 11 mm on the day of hCG. Dots represent subgroups with n≥50; circles represent smaller subgroups.
(DOCX)

S7 Fig Observed proportions and expected probabilities for severe OHSS associated with the E2 level on the day of hCG. Dots represent subgroups with n≥50; circles represent smaller subgroups.
(DOCX)

S1 Table Odds ratios for OHSS of any grade for the number of follicles ≥11 mm and the E2 level on the day of hCG. (DOCX)

S2 Table Diagnostic test characteristics for various rules to predict OHSS of any grade. (DOCX)

S1 Text Prediction of OHSS of any grade. (DOCX)
