# Supplementary material for: Prediction of Ovarian Hyperstimulation Syndrome in Patients Treated with Corifollitropin alfa or rFSH in a GnRH Antagonist Protocol
Source: PLoS One. 2016 Mar 7;11(3):e0149615. doi: 10.1371/journal.pone.0149615 (PMC4780699; doi:10.1371/journal.pone.0149615)
Supplement: S1 Fig — AUCs: 0.720 (follicles); 0.696 (E2); 0.744 (follicles and E2). (DOCX) [file pone.0149615.s001.docx]

**S1 Fig. ROC curves for the prediction of OHSS of any grade (136 cases) based on the number of follicles ≥ 11 mm on the day of hCG, the E_2_ level on the day of hCG or both.** AUCs: 0.720 (follicles); 0.696 (E_2_); 0.744 (follicles and E_2_).

**
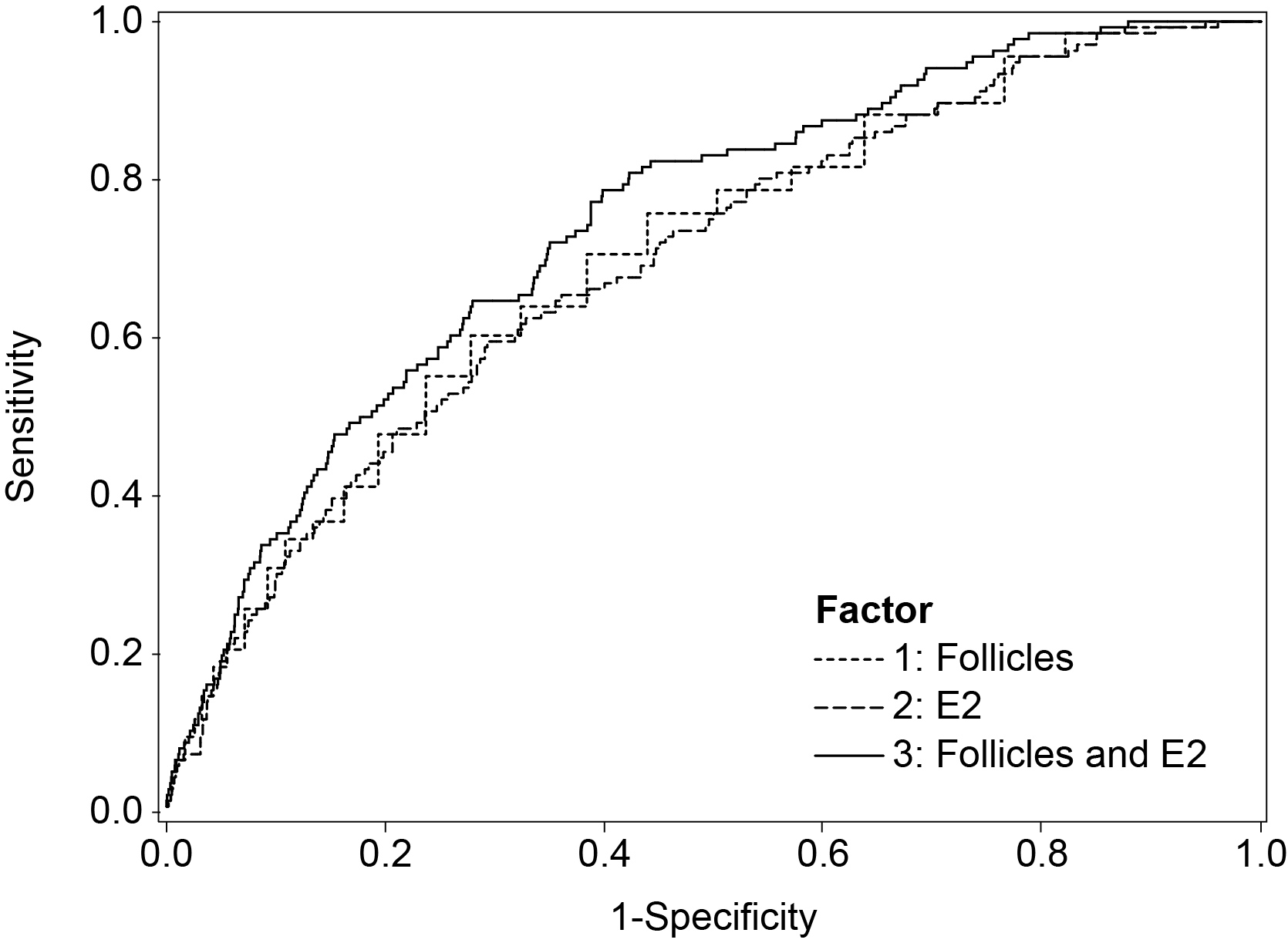
**
