# Supplementary material for: Prediction of Ovarian Hyperstimulation Syndrome in Patients Treated with Corifollitropin alfa or rFSH in a GnRH Antagonist Protocol
Source: PLoS One. 2016 Mar 7;11(3):e0149615. doi: 10.1371/journal.pone.0149615 (PMC4780699; doi:10.1371/journal.pone.0149615)
Supplement: S3 Fig — Dots represent subgroups with at least 50 subjects; circles represent smaller subgroups. (DOCX) [file pone.0149615.s003.docx]

**S3 Fig. Observed proportions and expected probabilities for OHSS of any grade associated with the E_2_ level on the day of hCG.** Dots represent subgroups with at least 50 subjects; circles represent smaller subgroups.

**
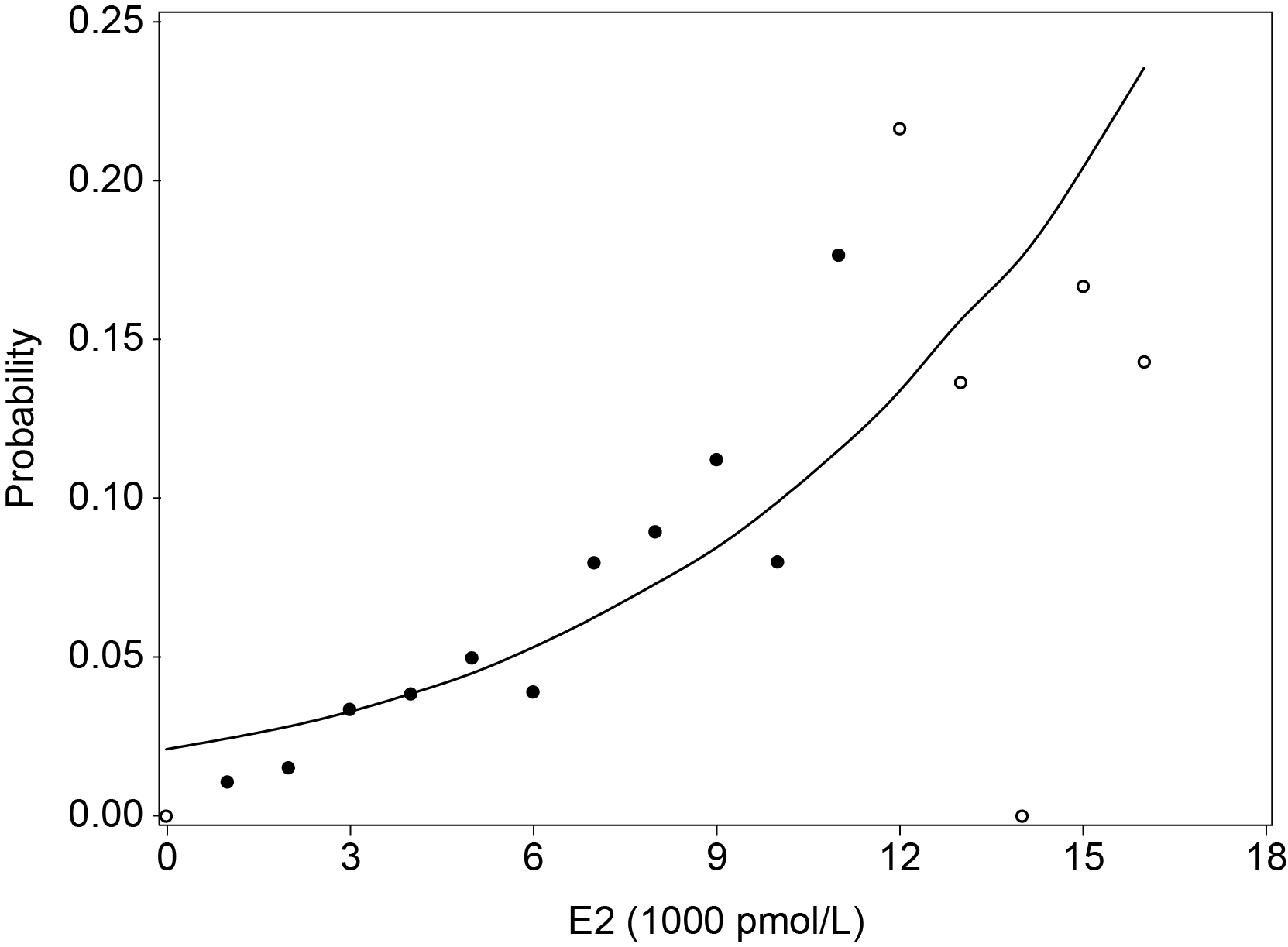
**
