# Supplementary material for: Prediction of Ovarian Hyperstimulation Syndrome in Patients Treated with Corifollitropin alfa or rFSH in a GnRH Antagonist Protocol
Source: PLoS One. 2016 Mar 7;11(3):e0149615. doi: 10.1371/journal.pone.0149615 (PMC4780699; doi:10.1371/journal.pone.0149615)
Supplement: S4 Fig — Dots represent subgroups with n≥50; circles represent smaller subgroups. (DOCX) [file pone.0149615.s004.docx]

**S4 Fig. Observed proportions and expected probabilities for moderate to severe OHSS associated with the number of follicles ≥ 11 mm on the day of hCG.** Dots represent subgroups with n≥50; circles represent smaller subgroups.

**
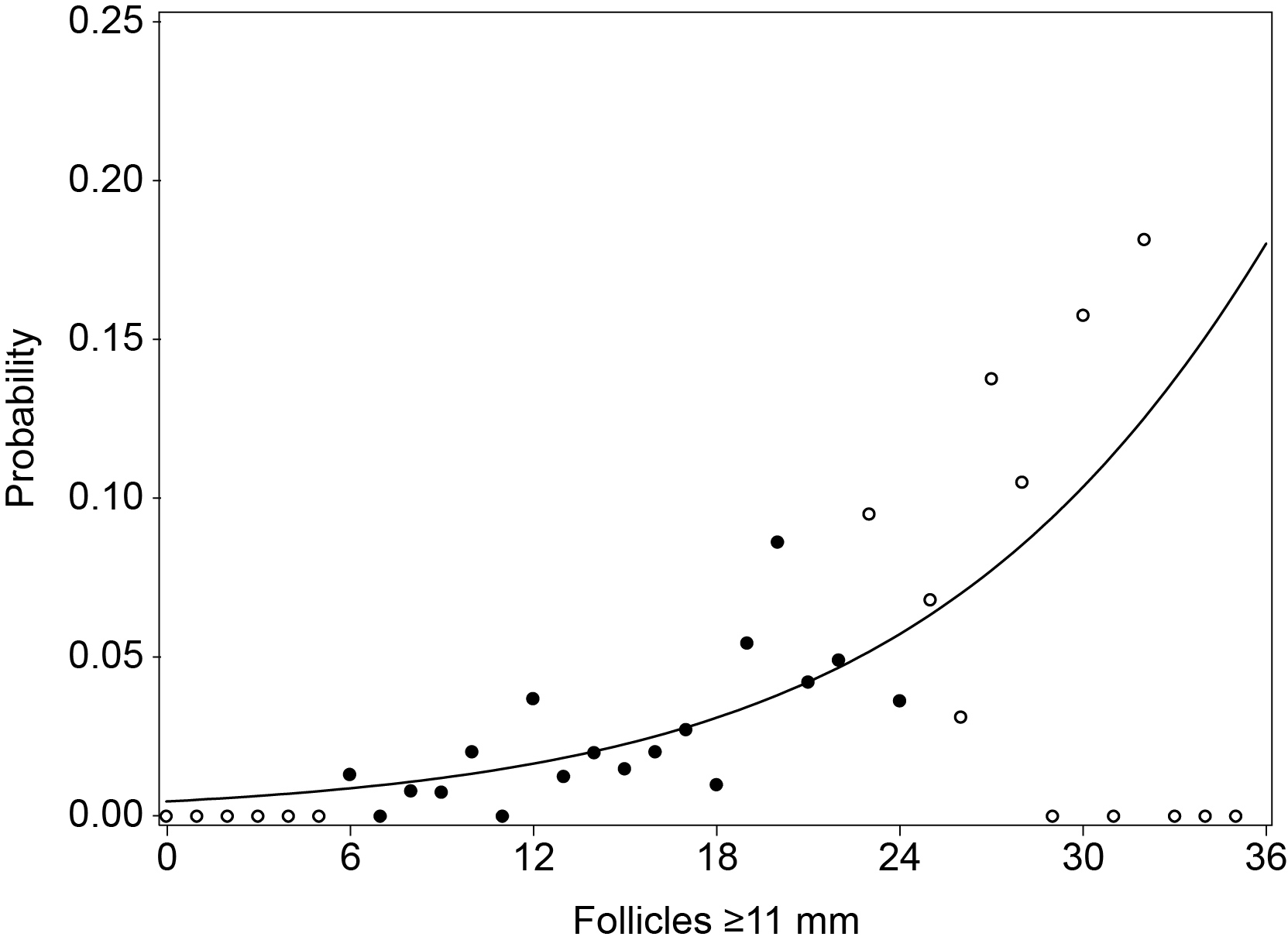
**
