# Supplementary material for: Prediction of Ovarian Hyperstimulation Syndrome in Patients Treated with Corifollitropin alfa or rFSH in a GnRH Antagonist Protocol
Source: PLoS One. 2016 Mar 7;11(3):e0149615. doi: 10.1371/journal.pone.0149615 (PMC4780699; doi:10.1371/journal.pone.0149615)
Supplement: S1 Table — (DOCX) [file pone.0149615.s008.docx]

**S1 Table.**  **Odds ratios for OHSS of any grade for the number of follicles ≥11 mm and the E_2_ level on the day of hCG.**

| **OHSS** | **Predictor(s)** | **Odds ratio*** | **95% CI** | **P-value** | **AUC**** |
| --- | --- | --- | --- | --- | --- |
| Any grade | Follicles ≥11 mm | 1.114 | 1.088, 1.140 | <0.0001 | 0.720 |
|  | E_2_ level | 1.183 | 1.136, 1.233 | <0.0001 | 0.696 |
|  | Follicles ≥11 mm  and E_2_ level | 1.109 | 1.059, 1.162 | <0.0001 | 0.744 |
