# Supplementary material for: Prediction of Ovarian Hyperstimulation Syndrome in Patients Treated with Corifollitropin alfa or rFSH in a GnRH Antagonist Protocol
Source: PLoS One. 2016 Mar 7;11(3):e0149615. doi: 10.1371/journal.pone.0149615 (PMC4780699; doi:10.1371/journal.pone.0149615)
Supplement: S2 Table — (DOCX) [file pone.0149615.s009.docx]

**S2 Table. Diagnostic test characteristics for various rules to predict OHSS of any grade.**

| OHSS | Predictor(s) | Sensitivity | Specificity | Predictive  Value | | Threshold |
| --- | --- | --- | --- | --- | --- | --- |
|  |  |  |  | Positive | Negative |  |
| Any grade | Follicles ≥11 mm | 70.6% | 61.6% | 9.8% | 97.3% | ≥16 |
|  | E_2_ level | 62.5% | 67.2% | 10.1% | 96.8% | ≥6200 |
|  | Follicles ≥11 mm and E_2_ level | 72.1% | 65.0% | 10.9% | 97.5% | ≥ -2.96^a^ |
