# Supplementary material for: Prediction of Ovarian Hyperstimulation Syndrome in Patients Treated with Corifollitropin alfa or rFSH in a GnRH Antagonist Protocol
Source: PLoS One. 2016 Mar 7;11(3):e0149615. doi: 10.1371/journal.pone.0149615 (PMC4780699; doi:10.1371/journal.pone.0149615)
Supplement: S1 Text — (DOCX) [file pone.0149615.s010.docx]

**S1 Text. Prediction of OHSS of any grade.**

In total, 136 of the 2433 subjects had OHSS of any grade (5.6%). The ROC curves for two predictors are shown in S1 Fig.

The AUC for the number of follicles ≥11 mm on the day of hCG was 0.720. The association with OHSS was statistically significant (P<0.0001). For every extra follicle the odds increased with a factor 1.11 (S1 Table). For the prediction of OHSS the optimal threshold was 16 follicles: women with 16 or more follicles ≥11 mm were at higher risk for OHSS than those with up to 15 follicles. The sensitivity and specificity were 70.6% and 61.6%, respectively. The percentage of women at ‘higher risk’ that actually experienced OHSS was 9.8% (positive predictive value, S2 Table).

The AUC for the E_2_ level on the day of hCG was 0.696. The association with OHSS was statistically significant (P<0.0001). For every 1000 pmol/L, the odds increased with a factor 1.18 (S1 Table). For the prediction of OHSS the optimal threshold was approximately 6200 pmol/L: women with an E_2_ level ≥ 6200 pmol/L were at higher risk for OHSS than those with an E_2_ <6200 pmol/L. In practice, a threshold of 6000 pmol/L (1634 pg/mL) may be used. The sensitivity and specificity were 62.5% and 67.2%, respectively. The percentage of women at ‘higher risk’ that actually experienced OHSS was 10.1% (positive predictive value, S2 Table).Compared to the number of follicles, E_2_ concentration was of lower prognostic value.

To determine whether the sensitivity of the OHSS prediction could be increased by combining the two factors, analysis was carried out in an ad hoc fashion by considering subjects with ≥16 follicles ≥11 mm or E_2_ ≥6200 pmol/L (or both) at higher risk for OHSS. However, the increase in sensitivity (83.8%, not shown) came at the price of a decreased specificity (50.4%, not shown). A more sophisticated way was to include both factors simultaneously in a model and construct a so-called linear predictor (S2 Table). The AUC of that model was 0.744 (S1 Fig., S1 Table).
